# In Planta Production of the Receptor-Binding Domain From SARS-CoV-2 With Human Blood Group A Glycan Structures

**DOI:** 10.3389/fchem.2021.816544

**Published:** 2022-02-01

**Authors:** Julia König-Beihammer, Ulrike Vavra, Yun-Ji Shin, Christiane Veit, Clemens Grünwald-Gruber, Yasmin Gillitschka, Jasmin Huber, Manuela Hofner, Klemens Vierlinger, Dieter Mitteregger, Andreas Weinhäusel, Richard Strasser

**Affiliations:** ^1^ Department of Applied Genetics and Cell Biology, Institute of Plant Biotechnology and Cell Biology, University of Natural Resources and Life Sciences, Vienna, Austria; ^2^ Core Facility Mass Spectrometry, University of Natural Resources and Life Sciences Vienna, Muthgasse, Austria; ^3^ Core Facility Mass Spectrometry, University of Natural Resources and Life Sciences, Vienna, Austria; ^4^ Laboratory Dr. Kosak, Dr. Reckendorfer, Vienna, Austria

**Keywords:** blood group antigen, carbohydrate, glycoengineering, glycosylation, posttranslational modification, virus

## Abstract

Glycosylation of viral envelope proteins is important for infectivity and immune evasion. The SARS-CoV-2 spike protein is heavily glycosylated and host-derived glycan modifications contribute to the formation of specific immunogenic epitopes, enhance the virus-cell interaction or affect virus transmission. On recombinant viral antigens used as subunit vaccines or for serological assays, distinct glycan structures may enhance the immunogenicity and are recognized by naturally occurring antibodies in human sera. Here, we performed an *in vivo* glycoengineering approach to produce recombinant variants of the SARS-CoV-2 receptor-binding domain (RBD) with blood group antigens in *Nicotiana benthamiana* plants. SARS-CoV-2 RBD and human glycosyltransferases for the blood group ABH antigen formation were transiently co-expressed in *N. benthamiana* leaves. Recombinant RBD was purified and the formation of complex N-glycans carrying blood group A antigens was shown by immunoblotting and MS analysis. Binding to the cellular ACE2 receptor and the conformation-dependent CR3022 antibody showed that the RBD glycosylation variants carrying blood group antigens were functional. Analysis of sera from RBD-positive and RBD-negative individuals revealed further that non-infected RBD-negative blood group O individuals have antibodies that strongly bind to RBD modified with blood group A antigen structures. The binding of IgGs derived from sera of non-infected RBD-negative blood group O individuals to blood group A antigens on SARS-CoV-2 RBD suggests that these antibodies could provide some degree of protection from virus infection.

## Introduction

Processing of glycans on viral proteins depends on the protein conformation and the glycosylation machinery of the expressing cell. Viral spike proteins are heavily glycosylated, and glycosylation is crucial for virus infection and the host immune response (Watanabe et al., 2020a; Li Q et al., 2020). During viral infection, enveloped viruses engage with host cell receptors to initiate uptake by the cells. SARS-CoV-2 cell entry is dependent on the heavily glycosylated spike protein. The spike protein is a type I transmembrane protein with a large N-terminal ectodomain that protrudes from the viral surface (Walls et al., 2020). The spike protein binds to the cell surface receptor angiotensin-converting enzyme 2 (ACE2) which mediates membrane fusion and virus entry (Hoffmann et al., 2020; Walls et al., 2020). The SARS-CoV-2 monomeric spike protein possesses 22 N-glycosylation sites (Asn-X-Ser/Thr, with X any amino acid except proline) and displays a mixture of oligomannosidic, hybrid-type and complex N-glycans on recombinantly produced protein (Watanabe et al., 2020a; Shajahan et al., 2021). Furthermore, recent studies have shown site-specific differences in N-glycan composition between recombinant spike and infectious virions obtained from different human cells (Turoňová et al., 2020; Brun et al., 2021) highlighting the importance of glycoprotein presentation/site accessibility, quaternary protein architecture, and the host glycosylation machinery.

**FIGURE 1:**
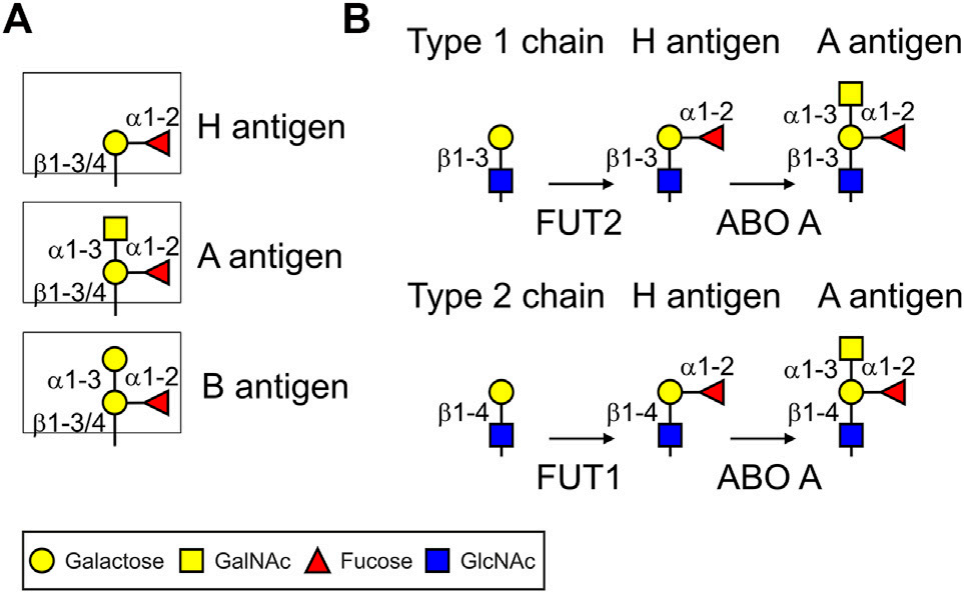
Cartoon illustration of ABH(O) blood group antigens and biosynthetic pathways. **(A)** ABH blood group carbohydrate structures. **(B)** Biosynthetic steps to produce blood group A type 1 and 2 antigens common to N- and O-glycans. FUT1/FUT2: α1,2-fucosyltransferases; ABO A: α1,3-GalNAc-transferase (A enzyme). For the biosynthesis of blood group B structures the ABO A enzyme is replaced by the α1,3-galactosyltransferase (ABO B enzyme).

The ABH(O) blood group antigens are specific carbohydrate structures attached to glycoproteins and glycolipids present on the surface of human erythrocytes, epithelial and endothelial cells of different tissues (Yamamoto, 2004; Cooling, 2015). Responsible for blood group A biosynthesis is an N-acetylgalactosaminyltransferase (ABO A enzyme) that transfers an N-acetylgalactosamine (GalNAc) in α1,3-linkage to a precursor structure called the H antigen (Fucα1,2-Galβ-R) resulting in the trisaccharide GalNAcα1-3-(Fucα1,2)-Galβ-R (Figure 1). By contrast, the ABO B enzyme transfers a galactose residue in α1,3-linkage to the H antigen resulting in the trisaccharide Galα1-3-(Fucα1,2)-Galβ-R. The ABO A and ABO B glycosyltransferases are encoded by distinct alleles of the ABO gene locus which are codominant to each other. The O allele is a null allele at the ABO locus and lacks the corresponding A or B glycosyltransferase activities, in which case the H antigen remains unmodified. Individuals who have the genotype AA or AO synthesize exclusively the A antigen, BB and BO individuals have blood group B and individuals with genotype OO have the blood group O. Blood group O individuals have high titers of circulating antibodies against A and B antigens (Stussi et al., 2005). Blood group A individuals have anti-B antibodies and blood group B individuals have anti-A antibodies. Blood group AB individuals express both antigens and lack anti-A or anti-B antibodies (Cooling, 2015). While the biological role of these carbohydrates is still poorly understood, the blood group antigens are of clinical relevance and anti-blood group antibodies are critical for blood transfusions and transplantation medicine.

Blood group frequencies vary among human populations and the specific exposure to pathogens may explain some of the observed variations in infectivity (Goel et al., 2021). Earlier epidemiological observations indicated that ABO blood groups may contribute to susceptibility to SARS-CoV-1 infection (Cheng et al., 2005). In a more recent genome-wide association study of nearly 2000 SARS-CoV-2 infected individuals, a gene cluster carrying the ABO locus was enriched in patients with COVID-19 (Ellinghaus et al., 2020). Furthermore, a study with 2,173 patients from different Chinese hospitals reported that ABO blood groups display different association risks for the infection with SARS-CoV-2 resulting in COVID-19 (Zhao J. et al., 2020). Blood group A was associated with an increased risk whereas blood group O was associated with a decreased risk. A meta-analysis reported that the proportion of blood group A in patients infected with SARS-CoV-2 was significantly increased compared to a control group (Li J et al., 2020). From these and further epidemiological studies using different populations the association between distinct ABO blood groups and COVID-19-linked hospitalization is well established (Leaf et al., 2020; Wu et al., 2020; Miotto et al., 2021). Overall, these studies suggest a role for blood group A glycans and anti-A antibodies in SARS-CoV-2 infection, which could potentially be harnessed for applications aiming to prevent the transmission from individuals to individuals. Still, the underlying mechanisms of increased risks for blood group A individuals are unclear and different hypotheses have been discussed and tested (Arend, 2021; Deleers et al., 2021; Goel et al., 2021; Wu et al., 2021). Potential mechanisms include a protective role of natural antibodies against blood group antigens (Arend, 2021; Deleers et al., 2021) or the presence of a lectin domain in RBD that mediates binding to blood group A structures on the cell surface of respiratory epithelial cells that could promote infection of the cells (Wu et al. 2021). There is an association of variations in angiotensin-converting enzyme-1 (ACE1) activity and ABO blood groups (Goel et al., 2021) and the half-life of coagulation factors like factor VIII and von Willebrand factor is altered by N-glycans carrying certain ABO blood group structures (Gallinaro et al., 2008). As a consequence, increased levels of von Willebrand factor in blood group A individuals could contribute to thrombosis and adverse outcomes upon SARS-CoV-2 infection. Based on these observations it is possible that more than one mechanism could protect blood group O individuals from infection and severe disease progression.

Here, we performed glycoengineering in *N. benthamiana* to produce betacoronavirus antigens furnished with blood group carbohydrate structures. We transiently expressed the receptor-binding domain (RBD) of the SARS-CoV-2 spike protein (RBD-215) (Shin et al., 2021) and the RBD from the SARS-CoV-1 spike in *N. benthamiana* and characterized the binding to antibodies and the cellular ACE2 receptor. The binding of IgGs derived from sera of blood group O and B donors to blood group A antigens on SARS-CoV-2 RBD suggests that these antibodies could provide some degree of protection from transmission of virus carrying blood group A carbohydrates.

## Results

### Recombinant RBD With Blood Group A N-Glycans can Be Produced in *N. benthamiana*

**FIGURE 2:**
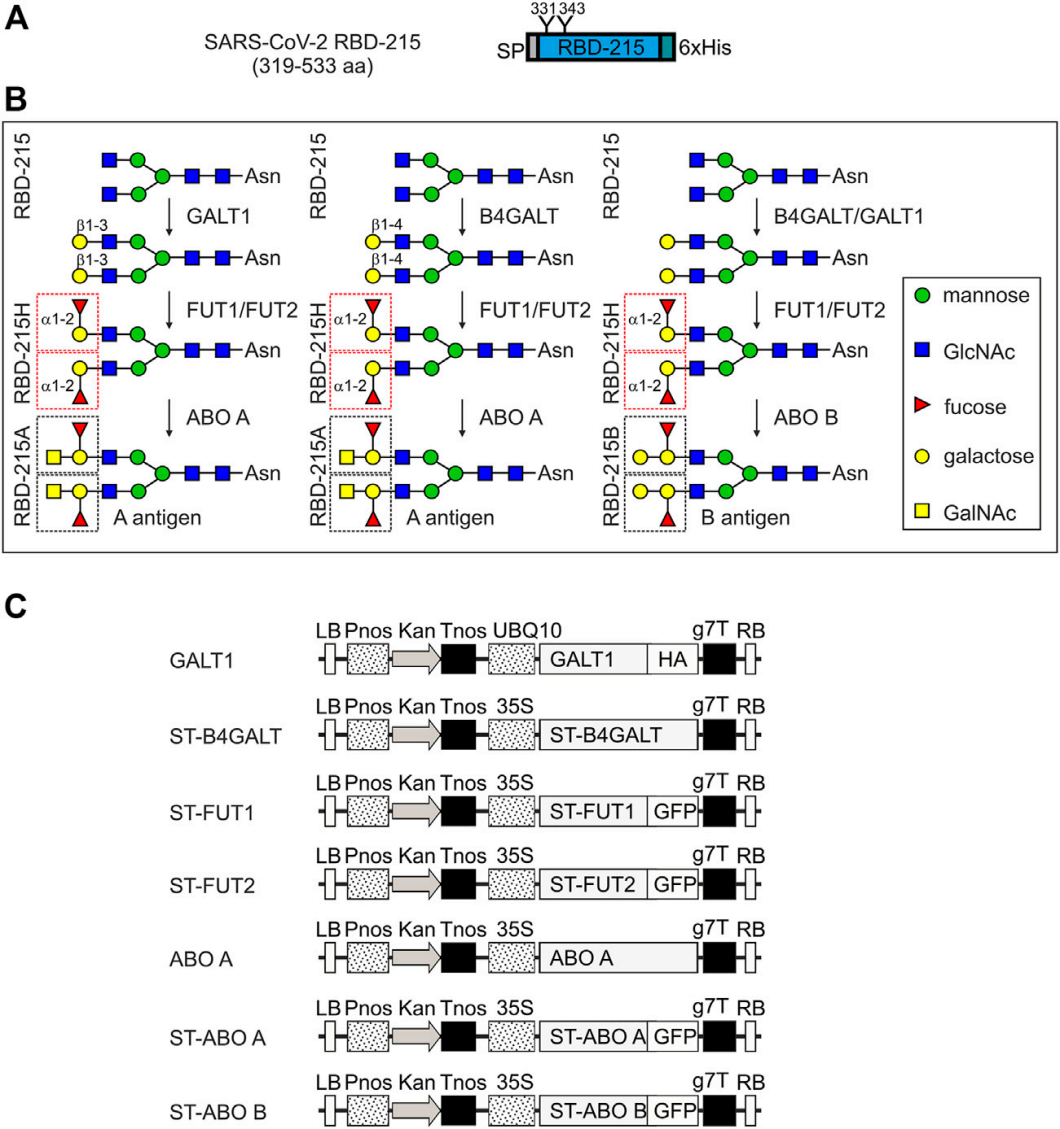
Schematic overview of the glycoengineering strategy and the used expression constructs. **(A)** Illustration of the plant-expressed RBD (amino acid region 319–533 of the SARS-CoV-2 spike protein). **(B)** Glycoengineering steps to produce different ABH blood group antigens in plants. GALT1: *A. thaliana* Lewis-type β1,3-galactosyltransferase 1; B4GALT: human β1,4-galactosyltransferase; FUT1/FUT2: human α1,2-fucosyltransferases. ABO A: human α1,3-GalNAc-transferase; ABO B: human α1,3-galactosyltransferase. **(C)** Schematic presentation of the expression cassettes for the different glycosyltransferases. LB: left border; Pnos: nopaline synthase gene promoter; Kan: neomycin phosphotransferase II gene; Tnos: nopaline synthase gene terminator; UBQ10: *A. thaliana* ubiquitin-10 promoter; ST: N-terminal *trans*-Golgi targeting region from rat α2,6-sialyltransferase (amino acids 1–52); HA: hemagglutinin tag; GFP: green fluorescent protein; g7T: agrobacterium gene 7 terminator; RB: right border.

Recombinant RBD-215 (amino acids 319–533 of the SARS-CoV-2 spike protein, Figure 2A) expressed in glycoengineered ΔXT/FT plants carries mainly GlcNAc_2_Man_3_GlcNAc_2_ (GnGn) N-glycans on both N-glycosylation sites (Shin et al., 2021). To see if these complex N-glycans can be modified with blood group carbohydrates, we transiently co-expressed glycosyltransferases for the formation of blood group antigens (Figure 2B). To achieve modification of recombinant RBD-215 with blood group A structures we co-expressed either human β1,4-galactosyltransferase (B4GALT) (Strasser et al., 2009) or *A. thaliana* β1,3-galactosyltransferase (GALT1) (Strasser et al., 2007), with one human α1,2-fucosyltransferase (FUT1 or FUT2), and the human ABO A enzyme transiently in ΔXT/FT *N. benthamiana*. To avoid interference from other N-glycan processing steps, the catalytic domain of B4GALT (type 2 chain formation) was targeted to the *trans*-Golgi using the N-terminal targeting sequence from rat α2,6-sialyltransferase (ST) (Boevink et al., 1998; Strasser et al., 2009). The catalytic domains of FUT1 and FUT2 were also fused to the ST-region to enable transfer of the fucose to the galactose in the same Golgi compartment. In addition, GFP was attached to the C-terminal end of the glycosyltransferase to enable monitoring of the subcellular localization (Figure 2C). *A. thaliana* GALT1 resides in the *trans*-Golgi (Strasser et al., 2007) and therefore the native sequence was expressed to achieve efficient β1,3-galactosylation (type 1 chain formation, Figure 1). The human ABO A enzyme was expressed as a chimeric protein with the catalytic domain fused to ST and GFP (ST-ABO A) and as a native enzyme without any foreign targeting signal or tag. ST-FUT1, ST-FUT2 and ST-ABO A expressed well in plants and displayed Golgi localization (Supplementary Figure S1).

**FIGURE 3:**
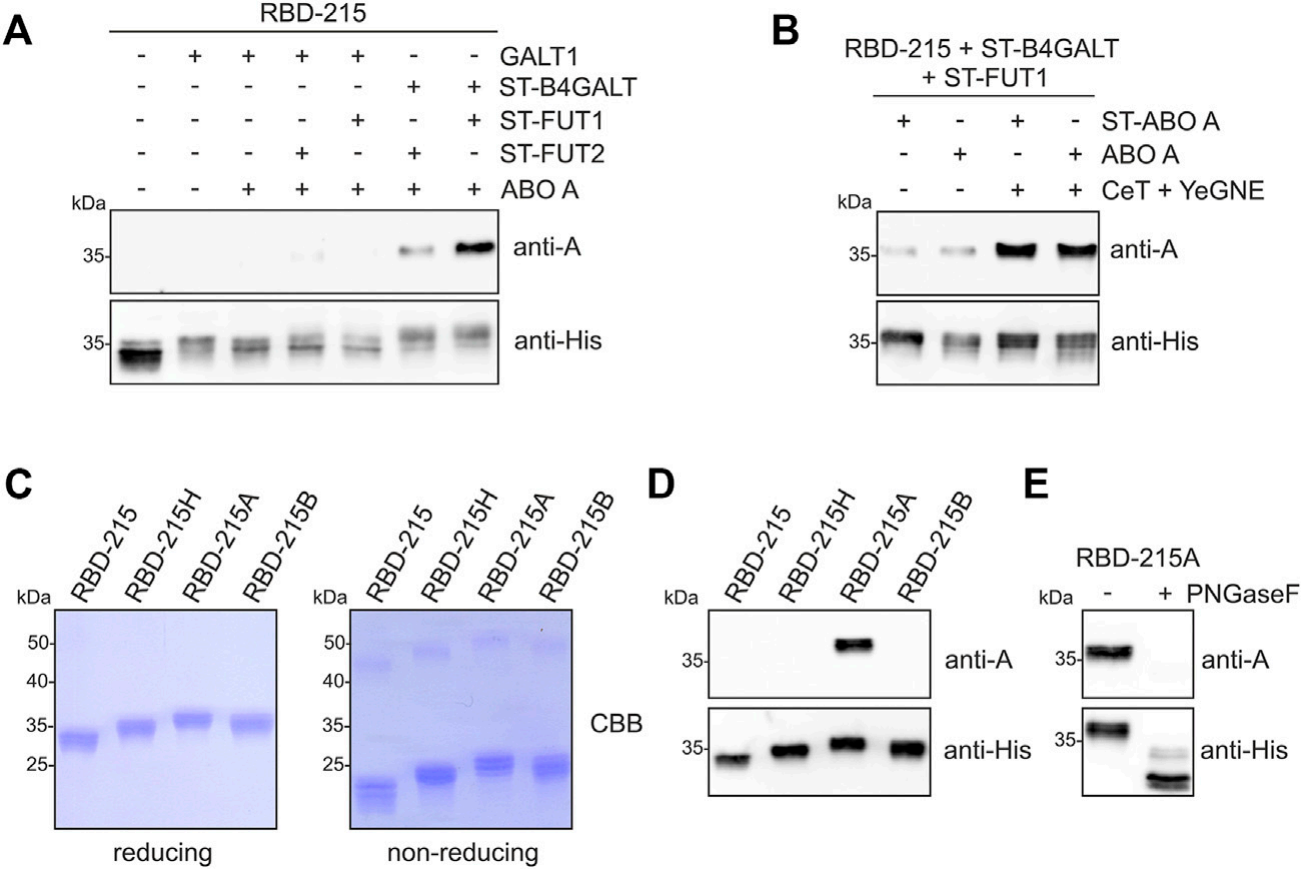
Plant-based production of RBD-215 with type 2 blood group A antigens. **(A)** RBD-215 was transiently expressed in the glycoengineered *N. benthamiana* line ΔXT/FT together with the indicated glycosyltransferases. 3 days-after infiltration, RBD-215 was purified from crude protein extracts using magnetic beads and subjected to SDS-PAGE, and immunoblotting with anti-blood group A (3-3A) or anti-His-tag antibodies. **(B)** Co-expression of *Y. enterocolitica* UDP-GlcNAc 4-epimerase (YeGNE) and *C. elegans* UDP-GlcNAc/UDP-GalNAc transporter (CeT) improves the formation of blood group A antigens. **(C)** RBD-215 variants were IMAC-purified from the apoplastic fluid of infiltrated line ΔXT/FT and subjected to SDS-PAGE under reducing and non-reducing conditions. **(D)** SDS-PAGE and immunoblotting of IMAC-purified RBD-215 variants with anti-blood group A (3-3A) or anti-His-tag antibodies. **(E)** PNGase F digestion of IMAC-purified RBD-215A.

RBD-215 co-expressed with the different glycosyltransferases was purified from crude protein extracts and subjected to SDS-PAGE and immunoblotting with blood group A-specific antibodies (Figure 3A; Supplementary Figure S1). Co-expression of ST-B4GALT, ABO A and either ST-FUT1 or ST-FUT2 (ST-FUT2 was less efficient than ST-FUT1) resulted in reactivity with the blood-group A-specific antibody suggesting the successful formation of type 2 chains. Detection of RBD-215 with the anti-His antibody resulted in a shift in mobility that was consistent with the N-glycan elongation mediated by the co-expressed blood group A-specific glycosyltransferases. Expression of ST-ABO A led to the formation of RBD-215 that reacted with the blood-group A-specific antibody to a similar extent indicating that ST-ABO A and native ABO A are both functional and targeted to a late Golgi compartment when transiently expressed in plants (Supplementary Figure S1, S2).

UDP-GalNAc, the nucleotide sugar for the ABO A glycosyltransferase, is not very abundant in plants (Daskalova et al., 2010). However, we have previously shown that O-glycan engineering in plants can be optimized by co-expression of a *Yersinia enterocolitica* UDP-GlcNAc 4-epimerase (YeGNE) capable of converting UDP-GlcNAc to UDP-GalNAc and a *Caenorhabditis elegans* UDP-GlcNAc/UDP-GalNAc transporter (CeT) for increased transport of the donor substrate into the Golgi lumen (Castilho et al., 2012). Therefore, we examined whether these proteins improve the biosynthesis of blood group A type 2 structures. Immunoblotting revealed that co-expression results in a stronger signal with the blood group A-specific antibody (Figure 3B). This was further confirmed using a different blood group A-specific antibody (Supplementary Figure S2). Our initial glycoengineering approach suggested that blood group A type 1 structures are not efficiently produced on RBD-215 (Figure 3A). However, when we expressed GALT1, ST-FUT2 and ST-ABO A together with YeGNE and CeT we could modify N-glycans on RBD-215 with blood group A type 1 chains (Supplementary Figure S3).

Next, we purified RBD-215 variants with different blood group structures from the apoplastic fluid by immobilized metal affinity chromatography (IMAC) and analyzed the purified proteins by SDS-PAGE under reducing and non-reducing conditions (Figure 3C). Since the blood group A type 2 structures were more efficiently generated than type 1 structures, we focused only on the characterization of the former. Under reducing conditions, the RBD-215 proteins migrated at the expected positions. Under non-reducing conditions, a faster migration was observed for all variants which is likely caused by the presence of four disulfide bonds leading to a more compact shape. Compared to RBD-215, reduced mobility was detected for all glycoengineered variants and the blood-group A-specific antibody reacted only with the purified RBD-215 protein that was co-expressed with the ABO A glycosyltransferase (RBD-215A, Figure 3D), but not with the RBD-215 co-expressed with glycosyltransferases for H (RBD-215H), and B (RBD-215B) antigen formation. Upon PNGase F digestion of RBD-215A, the reactivity with the blood-group A-specific antibody was completely lost showing the presence of the modification on N-glycans (Figure 3E).

**FIGURE 4:**
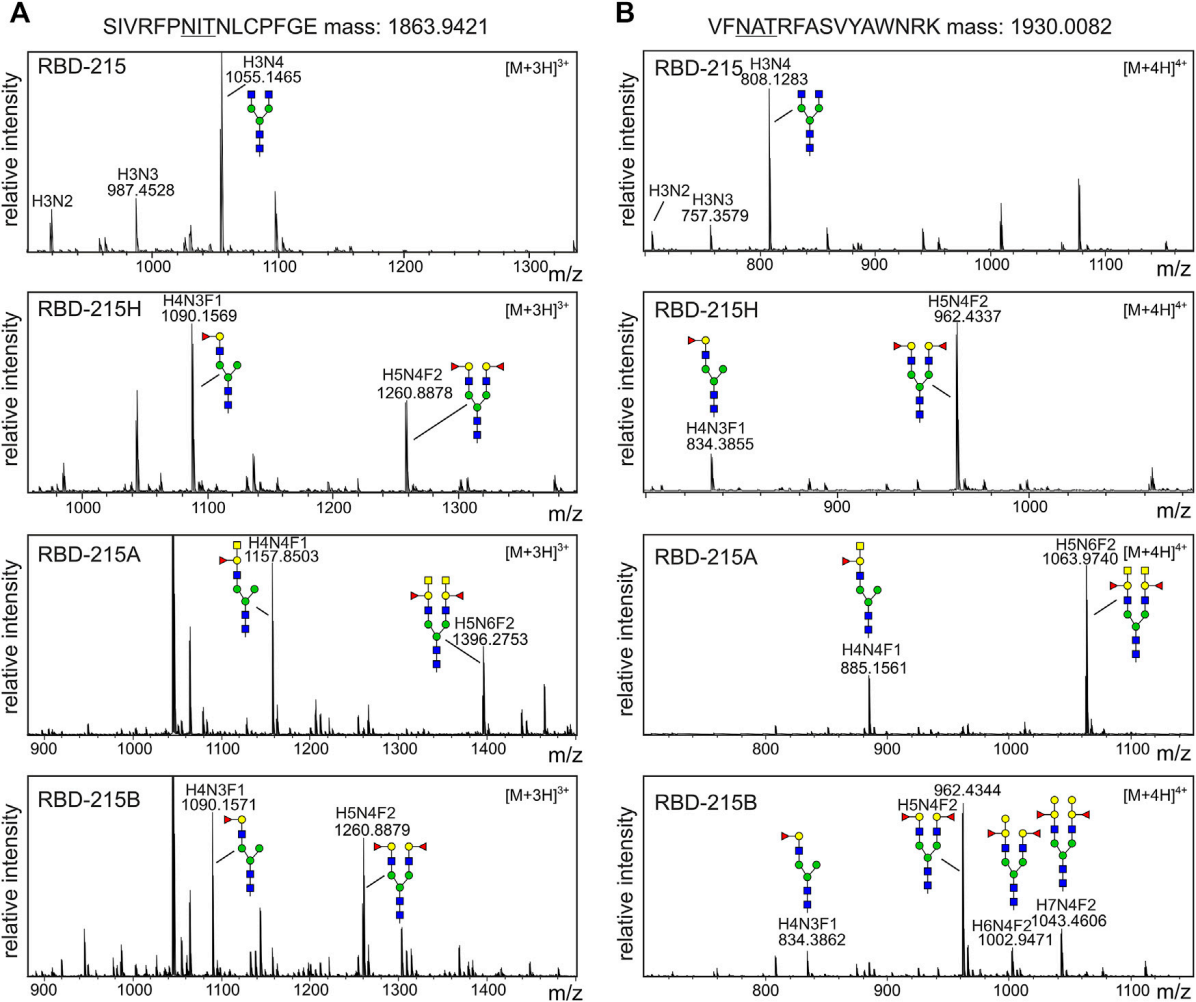
MS spectra of the RBD-215 glycopeptides carrying the N-glycosylation site N331 or N343. IMAC-purified RBD-215 variants were proteolytically digested and analyzed by MS. **(A)** Glycopeptides carrying N331 and **(B)** N343. Major N-glycan peaks are illustrated with a cartoon presentation (see Figure 2 for details). H: hexose; N: N-acetylhexosamine (HexNAc); F: fucose.

All four RBD-215 variants purified from the apoplastic fluid were proteolytically digested and the glycopeptides were analyzed by LC-ESI-MS. While RBD-215 carried mainly GnGn structures on both N-glycosylation sites, RBD-215H carried substantial amounts of mono- (major N-glycan at N331) and bi-antennary N-glycans (major N-glycan at N343) corresponding to H-type structures (Figures 4A,B; Supplementary Figure S4). RBD-215A that was obtained by additional co-expression of ST-ABO A displayed further elongation of the two N-glycan branches with an additional HexNAc residue. In line with the immunoblot data this indicates the successful formation of blood group A carbohydrate structures with terminal GalNAc. On the other hand, modification of H-type structures with galactose by co-expression of Golgi-targeted ST-ABO B (Supplementary Figure S1) was less efficient and only detected on the N-glycans at site N343 (Figure 4B). This could be either due to differences in catalytic activity of the ST-ABO B enzyme (Letts et al., 2006) or due to the removal of terminal galactose residues by galactosidases present in the apoplast (Kriechbaum et al., 2020). Taken together, this shows that blood group A structures can be efficiently produced on complex N-glycans of recombinant RBD-215 in *N. benthamiana* using a transient glycoengineering approach.

### RBD With Blood Group A Antigens Is Functional

**FIGURE 5:**
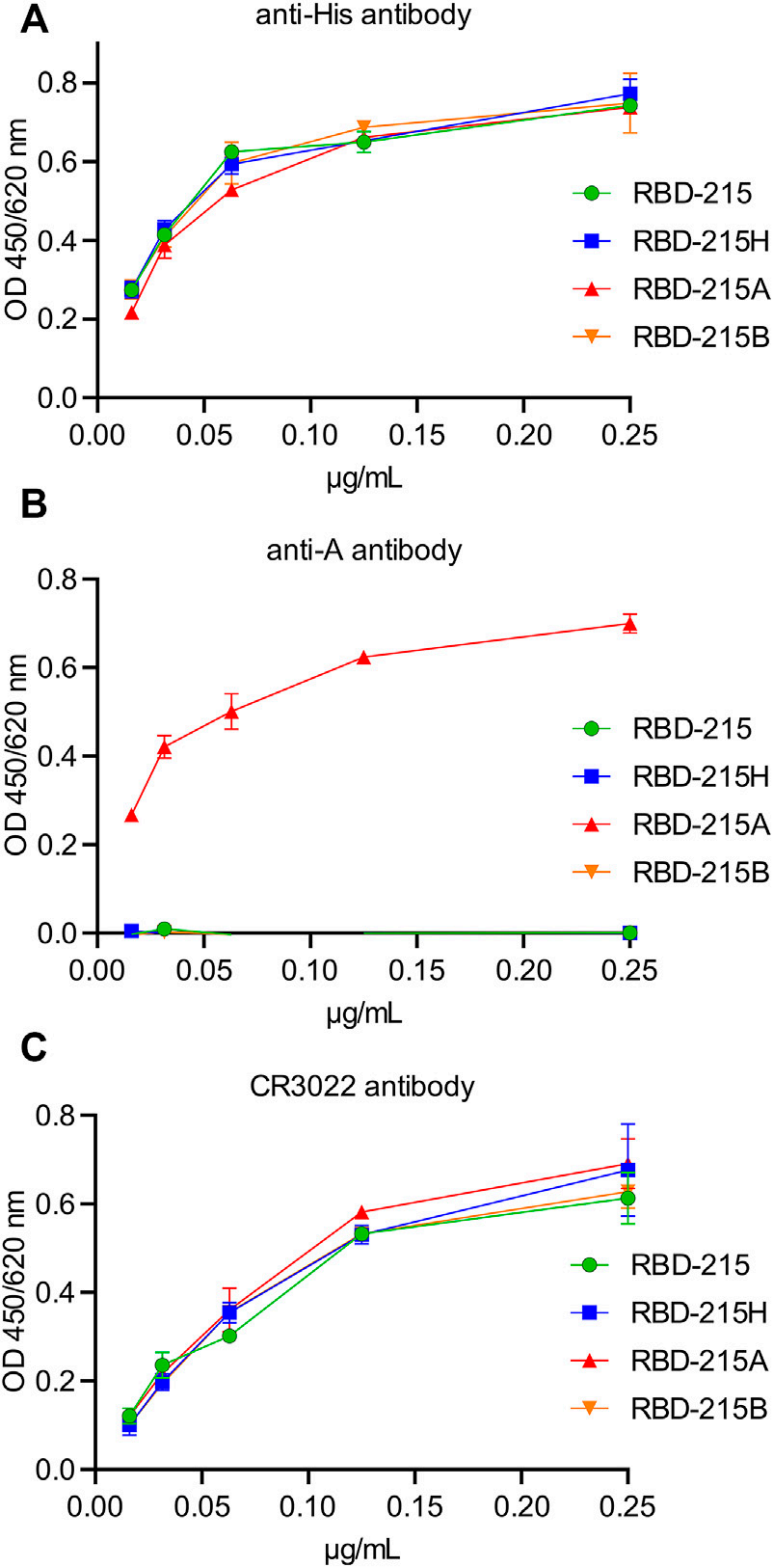
The anti-A antibody specifically reacts with RBD-215 carrying blood group A antigen structures. IMAC-purified RBD-215 variants were coated and ELISA was carried out with different concentrations of **(A)** antibodies against the His-tag, **(B)** the 3-3A antibody against anti-blood group A antigen structures and **(C)** CR3022, a conformation dependent anti-RBD antibody. Values represent the mean ± SD (*n* = 3).

The presence of distinct complex N-glycan modifications on RBD-215 may cause structural changes that affect the protein conformation. To assess this possibility ELISA was performed using a conformation-dependent RBD antibody. With the blood group A-specific antibody, binding was only detected with RBD-215A demonstrating the high specificity of the antibody (Figures 5A,B). The conformation-dependent RBD antibody CR3022 (Yuan et al., 2020) displayed comparable reactivity with all four variants suggesting that the N-glycan modifications do not have a major impact on the overall conformation of the viral antigen (Figure 5C).

**FIGURE 6:**
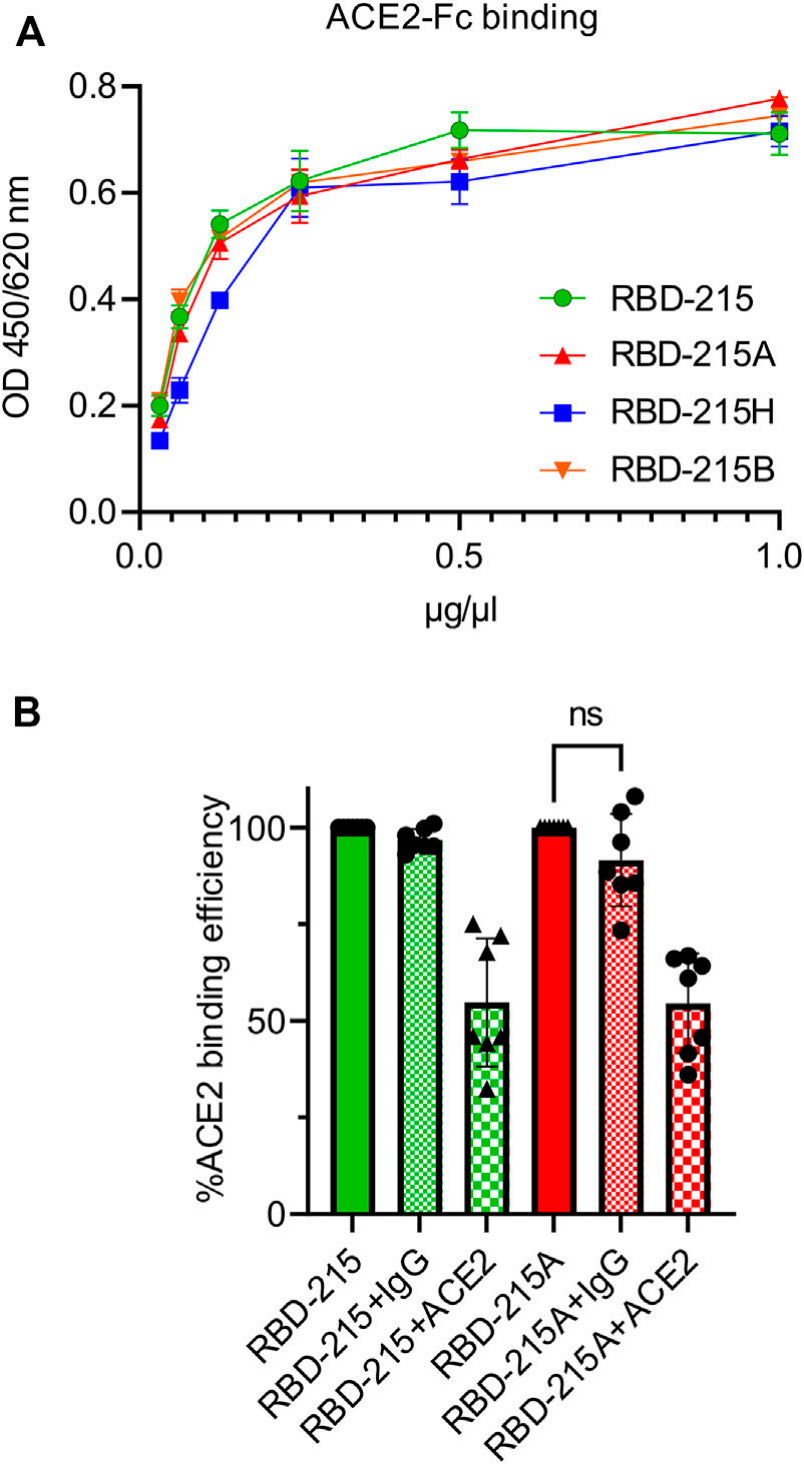
RBD-215 glycoforms display binding to ACE2-Fc. **(A)** ACE2-Fc binding ELISA. Binding curves of different concentrations of IMAC-purified RBD-215 glycoforms to plates coated with ACE2-Fc. Values represent the mean ± SD (n = 3). **(B)** ACE2-Fc competition ELISA. RBD-215 or RBD-215A were premixed with 3-3A anti-blood group A antibody (+IgG) or ACE2-Fc (+ACE2) and added to ELISA plates coated with ACE2-Fc. Detection was done with anti-His antibodies. Values represent the mean ± SD (*n* = 7). A student’s t-test was used to compare the difference in ACE2-Fc binding between RBD-215A and RBD-215A + IgG. “n.s.” not significant (*p* = 0.0870).

Binding of anti-blood group A antibodies to SARS-CoV-2 virus derived from blood group A positive cells could impair the interaction with the cellular receptor and subsequently the cell entry. We carried out an ACE2-binding ELISA to see if the presence of the blood group A-specific antibody interferes with binding of recombinant RBD-215 to immobilised ACE2-Fc. All four RBD-215 glycoforms showed binding to ACE2-Fc suggesting that N-glycan processing and formation of blood group carbohydrates does not interfere with the receptor binding (Figure 6A). Next, we carried out a competition ELISA to examine whether anti-blood group A-specific antibodies block interaction of RBD-215A and ACE2-Fc. As a control for the competition ELISA, we added a soluble ACE2-Fc that competes with the immobilized ACE2-Fc for binding (Gattinger et al., 2021). In the presence of soluble ACE2-Fc, reduced binding of RBD-215 and RBD-215A to ACE2-Fc was detected (Figure 6B). By contrast, the anti-blood group A antibody had only a minor effect on the RBD-215A ACE2-Fc interaction. This finding is consistent with previous studies reporting that the RBD N-glycans do not clash with the receptor-binding motif (RBM) on the SARS-CoV-2 spike (Pinto et al., 2020).

**FIGURE 7:**
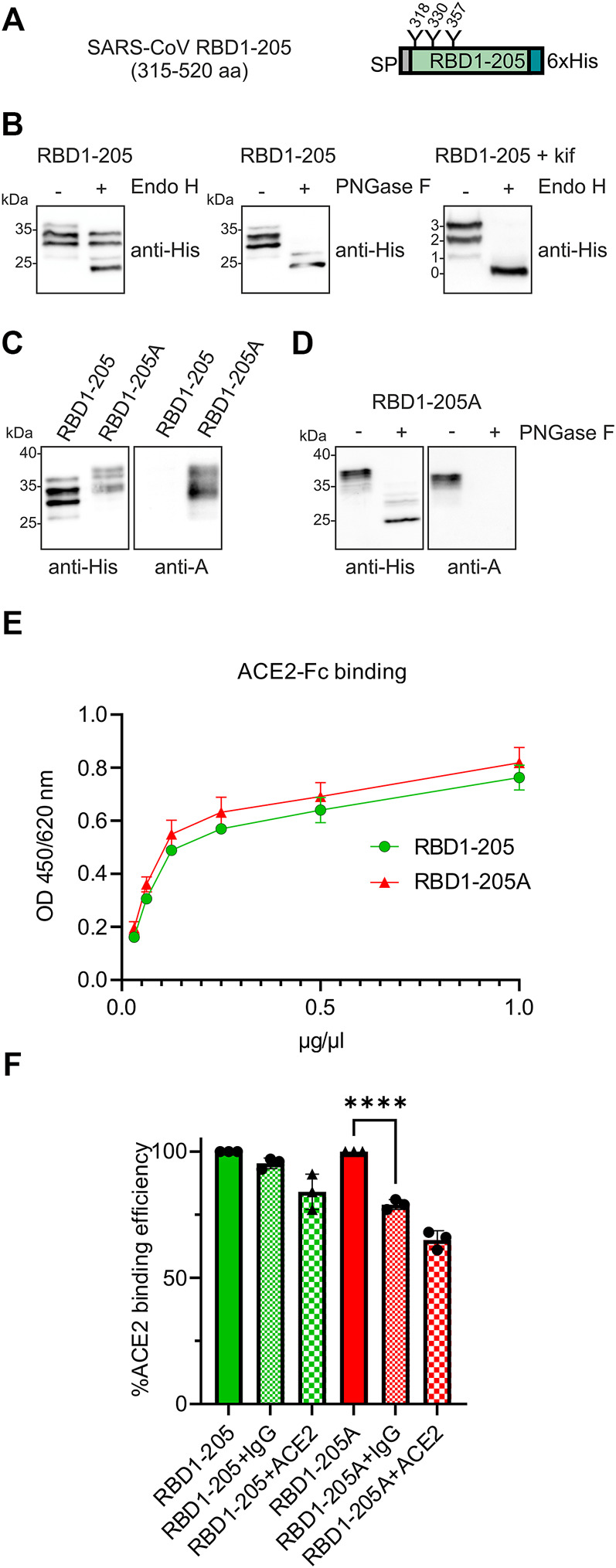
RBD1-205 N-glycans can be engineered to carry blood group A antigens. **(A)** Illustration of the plant-expressed RBD1-205 (amino acid region 315–520 of the SARS-CoV-1 spike protein). **(B)** Immunoblot analysis of IMAC-purified RBD1-205. Deglycosylation was done by digestion with Endo H or PNGase F. To produce RBD1-205 with Endo H-sensitive oligomannosidic N-glycans, RBD1-205 was expressed in the presence of the α-mannosidase inhibitor kifunensine (kif). 0, 1, 2, and 3 indicate the number of N-glycans present on RBD1-205. **(C)** Immunoblot analysis with anti-His and anti-blood group A (3-3A) antibodies **(D)** PNGase F digestion of RBD1-205. **(E)** ACE2-Fc binding ELISA. Binding of different concentrations of IMAC-purified RBD1-205 and RBD1-205A glycoforms to plates coated with ACE2-Fc. Values represent the mean ± SD (*n* = 3). **(F)** ACE2-Fc competition ELISA. RBD1-205 or RBD1-205A were premixed with the 3-3A anti-blood group A antibody (+IgG) or ACE2-Fc (+ACE2), and added to ELISA plates coated with ACE2-Fc. Detection was done with anti-His antibodies. Values represent the mean ± SD (*n* = 3). A student’s t-test was used to compare the difference in ACE2-Fc binding between RBD1-205A and RBD1-205A + IgG. “****” *p* < 0.0001.

To examine whether the same finding is observed for the RBD from SARS-CoV-1, we generated a recombinant protein carrying amino acids 315–520 of the SARS-CoV-1 spike protein (RBD1-205). In contrast to RBD-215, RBD1-205 from SARS-CoV-1 carries three N-glycosylation sites (Figure 7A). Purified RBD1-205 displayed several bands on immunoblots with the anti-His antibody. Deglycosylation of RBD1-205 with PNGase F or deglycosylation of an RBD1-205 variant carrying oligomannosidic N-glycans with Endo H revealed that the different bands are derived from incomplete occupancy of the three N-glycosylation sites (Figure 7B). The RBD1-205 variant was transiently expressed in *N. benthamiana* with or without the machinery (ST-GALT1, ST-FUT1, ST-ABO A, YeGNE, and CeT) for blood group A type 2 antigen formation. Like RBD-215, also RBD1-205 from SARS-CoV-1 could be modified with complex N-glycans that are recognized by the blood-group A-specific antibody on immunoblots (RBD1-205A, Figures 7C,D; Supplementary Figure S5) and RBD1-205 as well as RBD1-205A interacted with ACE2-Fc (Figure 7E). In contrast to RBD-215A, the blood-group A-specific antibody blocked the significantly engagement of RBD1-205A with ACE2-Fc (Figure 7F). This could be related to the presence of an additional N-glycan or the overall reduced affinity of the SARS-CoV-1 RBD for the ACE2 receptor.

### Blood Group A Antigen Structures Are Specifically Recognized by Natural Antibodies Present in Sera of Blood Group O and B Individuals

**FIGURE 8:**
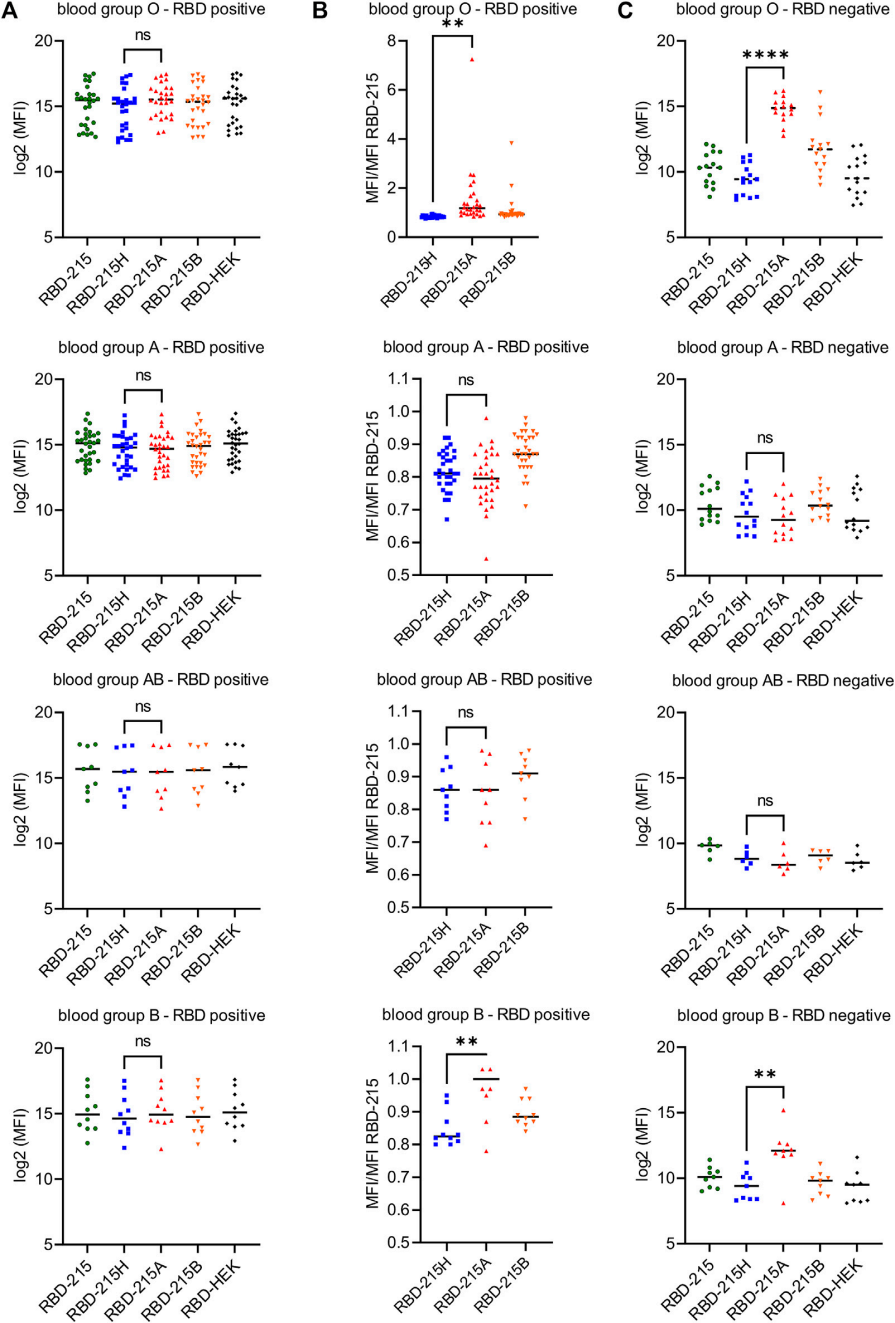
Sera from blood group O and B individuals react strongly with RBD-215A carrying blood group A antigens in a multiplex bead-based assay. **(A)** Reactivity of confirmed RBD positive sera (according to the WHO standard) to different RBD-215 glycoforms. RBD produced in HEK293 cells (RBD-HEK) was included for comparison. The line depicts the median in each group. A student’s t-test was used to compare the reactivity to RBD-215H (carrying the H antigen) and RBD-215A (carrying the A type 2 antigen). “n.s.” not significant; “**” *p* < 0.01; “****” *p* < 0.0001. Blood group O (*n* = 28), blood group A (*n* = 32), blood group AB (*n* = 9), blood group B (*n* = 10). **(B)** MFI values were normalized to the MFI values for RBD-215. The line depicts the median. A student’s t-test was used to compare the normalized values of RBD-215H and RBD-215A. **(C)** Reactivity of confirmed RBD negative sera (according to the WHO standard) to different RBD-215 glycoforms and RBD-HEK. Blood group O (*n* = 15), blood group A (*n* = 14), blood group AB (*n* = 6), blood group B (*n* = 9).

To examine whether natural antibodies present in sera from RBD-positive (SARS-CoV-2 exposed or vaccinated) and RBD-negative individuals differentially react with RBD-215 variants produced in plants using our glycoengineering approach, we carried out a multiplex bead-based assay with coupled recombinant viral antigens (Klausberger et al., 2021; Schwestka et al., 2021). In the RBD-positive cohorts, a comparison of the signal intensity did not reveal significant differences among the RBD-215 variants (Figure 8A). However, when the IgG binding intensity to the RBD-215 variants with blood group antigen structures was normalized to the signal from the unmodified RBD-215, a significant difference was revealed between RBD-215H and RBD-215A in the blood group O and B cohorts (Figure 8B; Supplementary Table S1). In line with this finding, sera from blood group O and B individuals showed significantly enhanced reactivity in the RBD-negative cohorts. In some RBD-negative sera with blood group O, the IgG binding signal was in the same range as observed for RBD-positive sera (Figure 8C) suggesting that those sera could provide enhanced protection for transmission of SARS-CoV-2 or similar coronaviruses from blood group A donors.

## Discussion

Glycosylation of viral proteins is not only important for protein quality control and folding (Margolin et al., 2020; Shin et al., 2021), but also for shielding of epitopes (Watanabe et al., 2020b) and interaction with cellular receptors (Hoffmann et al., 2021). Specific interference with receptor binding will lead to novel strategies to prevent infection of cells and tissues. In an earlier study it was shown that human anti-A antibodies inhibited the adhesion of SARS-CoV-1 spike to ACE2-expressing cells (Guillon et al., 2008). The recombinant SARS-CoV-1 spike protein was produced in engineered mammalian cells capable of blood group A biosynthesis and ACE2-expressing Vero cells used in the cell-based assay did not express the blood group A antigen. It is therefore plausible that anti-A antibodies can bind to blood group A antigens present on the heavily glycosylated SARS-CoV-1 spike protein. Our data for recombinant RBD1-205 with blood group A structures are in line with these data. The binding of anti-glycan antibodies could block the spike ACE2 interaction and protect against viral transmission from blood group A individuals to blood group O or B individuals. In analogy to SARS-CoV-1, the increased susceptibility of distinct blood groups to SARS-CoV-2 infection could be related to the presence of naturally circulating anti-A antibodies, which interfere with or even inhibit the virus–cell adhesion process and the subsequent transmission of the virus (Breiman et al., 2020; Goel et al., 2021). Such anti-glycan antibodies are considered part of the innate immune system to combat different bacterial and viral pathogens that carry specific glycan antigens (Cooling, 2015; Galili, 2020). The potential role in anti-viral activity of anti-blood group antibodies is already known for some time, for example, for anti-A antibody-mediated neutralization of HIV (Arendrup et al., 1991). Our data show that the presence of the blood group A antigen on the RBD N-glycans from SARS-CoV-2 does not significantly interfere with ACE2 receptor binding. Overall, this is not surprising because the N-glycosylation sites do not overlap with the amino acids involved in ACE2 binding (Piccoli et al., 2020; Yan et al., 2020). The absence of a major effect in the competition assay does not rule out the possibility that other N- or O-glycans with blood group A antigens on the SARS-CoV-2 spike protein have an impact on ACE2 binding. Alternatively, the binding of the anti-carbohydrate antibodies to viral glycans may cause cross linking or affect the protein conformation and dynamics as has been suggested for the SARS-CoV-2 neutralizing antibody S309 (Pinto et al., 2020). S309 is a non-RBM binding antibody that is targeted to an epitope containing the N-glycan attached at N343. On RBD-215 this N-glycosylation site was also highly modified with blood group A antigen structures and anti-A antibodies binding to the N-glycan at N343 may be implicated in virus neutralization by a similar mechanism as described for S309. Importantly, sotrovimab, a derivative of S309 has recently received an emergency use authorization (EUA) from the FDA for preventing severe COVID-19 disease and sotrovimab/S309 shows neutralization against evolving variants like Omicron which highlights the potential of these group of antibodies (Cameroni et al., 2021; Corti et al., 2021).

The SARS-CoV-2 spike protein monomer harbours 22 N-glycosylation sites that are all to a large extent glycosylated (Watanabe et al., 2020a; Zhao P. et al., 2020; Shajahan et al., 2021). In addition, more than 30 O-glycosylation sites have been predicted and some of them experimentally confirmed on recombinantly expressed spike protein variants including RBD (Zhao P. et al., 2020; Bagdonaite et al., 2021; Brun et al., 2021; Shajahan et al., 2021). Hence, there are numerous potential N- and O-glycosylation sites for the formation of blood group A antigens. So far, the studies focusing on the spike protein glycosylation in human cells did not provide evidence for the presence of H-, A- or B-type structures. However, this is not surprising as the used expression systems for recombinant viral proteins (HEK293, CHO, and insect cells) and host cells used for virus infection assays (e.g., Vero cells) lack the corresponding glycosyltransferase activities (Guillon et al., 2008; Lindberg et al., 2013; El Jellas et al., 2018). Therefore, it is essential to use cell lines or tissue organoids capable of producing a diverse range of glycans to study the contribution of individual modifications to virus infection or immunogenicity. In addition to the native glycosylation capacity of the used cells, virus infection may lead to a transcriptional reprogramming of the host cell resulting in the induction of glycosyltransferase expression that can modulate the cell-surface and viral glycans as has been recently shown for HIV infection (Colomb et al., 2020). All these possibilities must be considered when studying the impact of glycans on viral infection and transmission.

The current SARS-CoV-2 pandemic very drastically shows that we are not well equipped to cope with such an outbreak and numerous efforts are necessary to fight pathogens and increase the preparedness for future emerging or re-emerging viral threats. Recombinant anti-blood group antibodies (e.g., against blood group A antigen structures) could be used as protective drugs that are immediately available to fight newly emerging viruses, especially those that are heavily glycosylated, and carry a glycan shield for immune evasion (Watanabe et al., 2020b). Such recombinant anti-A antibodies could be administered in large quantity via nasal delivery as IgA or IgM formats (Ku et al., 2021) to blood group O or B individuals with low natural antibody levels. This may contribute to prevent initial virus uptake and thus reduce the spread of newly emerging respiratory viruses in populations until more specific drugs are developed.

## Materials and Methods

### Cloning of Expression Vectors

The generation of the pEAQ-*HT* expression vector for RBD-215 (SARS-CoV-2 RBD) was described previously (Shin et al., 2021). For RBD1-205 (SARS-CoV-1 RBD) expression, a synthetic DNA fragment (GeneArt, Thermo Fisher Scientific) coding for the barley α-amylase signal peptide fused to the N-terminal end of the spike domain (amino acids 315–520), and a 6x-histidine tag at the C-terminal end was cloned into the *Age*I/*Xho*I sites of pEAQ-*HT* (Sainsbury et al., 2009). Expression constructs for GALT1 (p43-GALT1) (Schwestka et al., 2021), ST-B4GALT (ST-GalT) (Strasser et al., 2009), *C. elegans* UDP-GlcNAc/UDP-GalNAc transporter (CeT: pH7WG2:GT), and *Y. enterocolitica* UDP-GlcNAc 4-epimerase (YeGNE: pH7WG2:GE) (Castilho et al., 2012) were available from previous studies. The ST-FUT1 expression vector was generated by cloning of a synthetic DNA fragment coding for a ST-FUT1 chimeric protein (amino acids 1–52 from rat α2,6-sialyltransferase fused to amino acids 37–365 from human FUT1) into p20F (Strasser et al., 2007) to generate p20-ST-FUT1. For ST-FUT2, the FUT2 coding sequence (amino acids 33–343 from human FUT2) was inserted into the *Bam*HI site of p20-ST to generate p20-ST-FUT2. The expression vector (pPT2M-ABO-A) for the full-length untagged ABO A transferase was generated by insertion of a synthetic codon-optimized fragment of the human ABO A coding region into the *Xba*I/*Bam*HI sites of pPT2M (Strasser et al., 2005). For the ST-ABO A expression vector, the coding region for amino acids 47–354 of human ABO A was cloned into the *Bam*HI site of p20-ST and for ST-ABO B the coding region for amino acids 47–354 of human ABO B was cloned in the same manner into p20-ST.

### Protein Expression and Purification

Syringe-mediated agroinfiltration of leaves from 5-week-old *N. benthamiana* ΔXT/FT (Strasser et al., 2008) was used for transient expression of RBD-215 variants. For the generation of blood group antigens, the enzymes were co-expressed by mixing of agrobacteria prior to agroinfiltration. For RBD-215 purification from crude extracts, leaves were harvested 3 days after infiltration, frozen in liquid nitrogen and homogenized using metallic beads and a mixer mill (Retsch). The homogenized material was resuspended in 3 volumes of 20 mM Na_2_HPO_4_, 100 mM NaCl (pH 7.4). Upon centrifugation to remove insoluble material, NaCl and imidazole was added to adjust the buffer to 20 mM Na_2_HPO_4_, 500 mM NaCl, and 10 mM imidazole (pH 7.4). His-tagged RBD-215 was purified using 100 µl His Mag Sepharose^®^ Ni (Cytiva) according to the manufacturer’s instructions. Bound proteins were eluted with 50 µl 20 mM Na_2_HPO_4_, 500 mM NaCl, and 500 mM imidazole (pH 7.4), mixed with sample loading buffer and used for SDS-PAGE and immunoblotting. For large scale purification of His-tagged proteins via immobilized metal affinity chromatography (IMAC), apoplastic fluid was collected from infiltrated leaves by low-speed centrifugation as described previously (Schwestka et al., 2021). After filtration through a 0.45 μm membrane filter (Merck Millipore), collected apoplastic fluid containing His-tagged RBD-215 in 500 mM NaCl, 20 mM Na_2_HPO_4_ and 10 mM imidazole (pH 7.4) was purified using a 1 ml HisTrap FF column (Cytiva) and the ÄKTA pure chromatography system (Cytiva). After washing with 15 column volumes and buffer containing 40 mM imidazole, bound proteins were eluted with 20 column volumes and buffer containing 250 mM imidazole. Fractions containing the protein of interest were pooled and dialyzed overnight against phosphate-buffered saline (PBS, pH 7.4) using SnakeSkin dialysis tubing (Thermo Fisher Scientific) with a 10 kDa molecular mass cutoff. Protein samples were then further concentrated using 10 kDa Amicon Ultra centrifugal filters (Merck Millipore). Purification of RBD-His (RBD-HEK) and human soluble ACE2-Fc from HEK293 cells has been described recently (Klausberger et al., 2021).

### Immunoblot Analysis

Purified proteins were subjected to SDS-PAGE under reducing or non-reducing conditions. Samples to be analyzed under non-reducing conditions were not boiled prior to loading. Separated proteins were either stained with Coomassie Brilliant Blue (Sigma-Aldrich) or transferred to a nitrocellulose membrane (Cytiva) and detected using anti-His (Thermo Fisher Scientific), anti-blood group A (3-3A—IgG antibody, Novus Biologicals), anti-blood group A (Z2A—IgM antibody, Santa Cruz Biotechnology), and JIM84 (Strasser et al., 2007) antibodies. For deglycosylation, proteins were denatured and incubated with or without Endo H or PNGase F (both from NEB) according to the manufacturer’s instructions.

### Liquid Chromatography-Electrospray Ionization-Mass Spectrometry (LC-ESI-MS).

Purified RBD-215 proteins were S-alkylated with iodoacetamide and digested in solution with endoproteinases LysC (Roche) and GluC (Promega). Digested samples were analyzed using a maXis 4G QTOF mass spectrometer (Bruker) as described (Klausberger et al., 2021).

### RBD and ACE2-Fc Binding ELISA

ELISA was carried out as described in detail recently (Schwestka et al., 2021). Briefly, 96-well plates (Nunc MaxiSorp™, Thermo Fisher Scientific) were coated overnight at 4°C with the indicated concentrations of purified RBD or ACE2-Fc proteins in PBS. For RBD-215 or RBD1-205 detection, plates were incubated for 2 h with the mouse-anti-His antibody (Thermo Fisher Scientific), anti-RBD antibody CR3022 (Klausberger et al., 2021; Shin et al., 2021) or anti-blood group A antibody (3-3A, Novus Biologicals). Plates incubated with anti-His or anti-blood group A antibody were washed again and incubated for 1 h with anti-mouse-HRP antibody (Sigma-Aldrich). CR3022 binding was analyzed using anti-human IgG (H + L)-HRP antibody (Promega). For detection, 150 µl/well of a 3,3′,5,5′-tetramethylbenzidin (TMB, Sigma-Aldrich) solution was added (1:60 of 0.4% TMB, 1:300 of 0.6% H_2_O_2_ in 50 mM phosphate-citrate buffer pH 5.0) and the reaction was stopped with 2 M H_2_SO_4_ (50 µl/well). The absorbance was measured at 450 nm using a TECAN Spark plate reader. Background resulting from unspecific binding of detection antibodies was subtracted from the obtained values. Three technical replicates were performed. Data were analyzed using GraphPad Prism Version 9.1.1.

### ACE2 Competition ELISA

For the ACE2 competition ELISA assay, 150 ng/well of ACE2-Fc in 50 µl PBS was coated onto F69 MaxiSORP Nunc-Immuno plates (Thermo Fisher Scientific) overnight at 4°C. After washing with PBS supplemented with 0.1% (v/v) Tween-20 (PBST), the plates were blocked with 1% (w/v) BSA in PBST for 1 h at room temperature. For the pre-incubation, 35 µl of 0.25 μg/ml RBD variants were mixed with 35 µl 1% (w/v) BSA in PBST, 35 µl 12.5 μg/ml anti-blood group A antibody (3-3A, Novus Biologicals) or 35 µl 14 μg/ml ACE2-Fc and incubated for 30 min at room temperature. Subsequently, 50 µl of pre-incubated sample mix was transferred onto the ACE2-Fc coated MaxiSORP plates and incubated for 2 h at room temperature. His-tagged RBD samples were detected with biotinylated mouse-anti-His antibody (Invitrogen), followed by incubation with a streptavidin-HRP conjugate (Roche). The chromogenic signal was developed using TMB as a substrate solution and analyzed as described for the binding ELISA.

### Luminex Assays

The RBD-215 variants and RBD-HEK were separately coupled to MagPlex carboxylated polystyrene microspheres (Luminex Corporation) and coupled glycoforms were then assayed in parallel with sera from different individuals collected at the AIT (AIT cohort). Coupling as well as Luminex assays were performed according to the manufacturer’s instructions with minor modifications as described in detail recently (Klausberger et al., 2021). AIT cohort comprises samples collected for routine SARS-CoV-2 serodiagnosis from 111 SARS-CoV-2 infected, uninfected and/or vaccinated individuals. Seronegativity or seropositivity has been determined via an eight-plex Luminex-based serotest and was based on cut-off values and end-point titers defined according to Frey et al., (1998) on the basis of 160 pre-COVID-19 sera.

## Data Availability

The raw data supporting the conclusion of this article will be made available by the authors, without undue reservation.
