# Supplementary material for: In Planta Production of the Receptor-Binding Domain From SARS-CoV-2 With Human Blood Group A Glycan Structures
Source: Front Chem. 2022 Feb 1;9:816544. doi: 10.3389/fchem.2021.816544 (PMC8846405; doi:10.3389/fchem.2021.816544)
Supplement: Supplementary file 1 [file DataSheet1.pdf]

Supplementary Material

In planta production of the receptor-binding domain from SARS-CoV-2 with human blood group glycan structures

Julia König-Beihammer[1], Ulrike Vavra[1], Yun-Ji Shin[1], Christiane Veit[1], Clemens Grünwald-Gruber[2], Yasmin Gillitschka[3], Jasmin Huber[3], Manuela Hofner[3], Klemens Vierlinger[3], Dieter Mitteregger[4], Andreas Weinhäusel[3], Richard Strasser[1,*]

Contents

Figure S1. Glycosyltransferase fusion proteins localize to the Golgi apparatus.

Figure S2. ABO and ST-ABO A display comparable activity when transiently co-expressed *in N. benthamiana* leaves.

Figure S3. RBD-215 carrying blood group A type 1 antigens (RBD-215A-type 1) can be successfully produced in *N. benthamiana*.

Figure S4. Quantification of RBD-215 glycopeptides.

Figure S5. Mass spectrometry (MS)-analysis of RBD1-205 carrying blood group A type 2 N-glycans.

Table S1. MFI values showing the binding of IgG from sera to RBD-215 glycoforms.

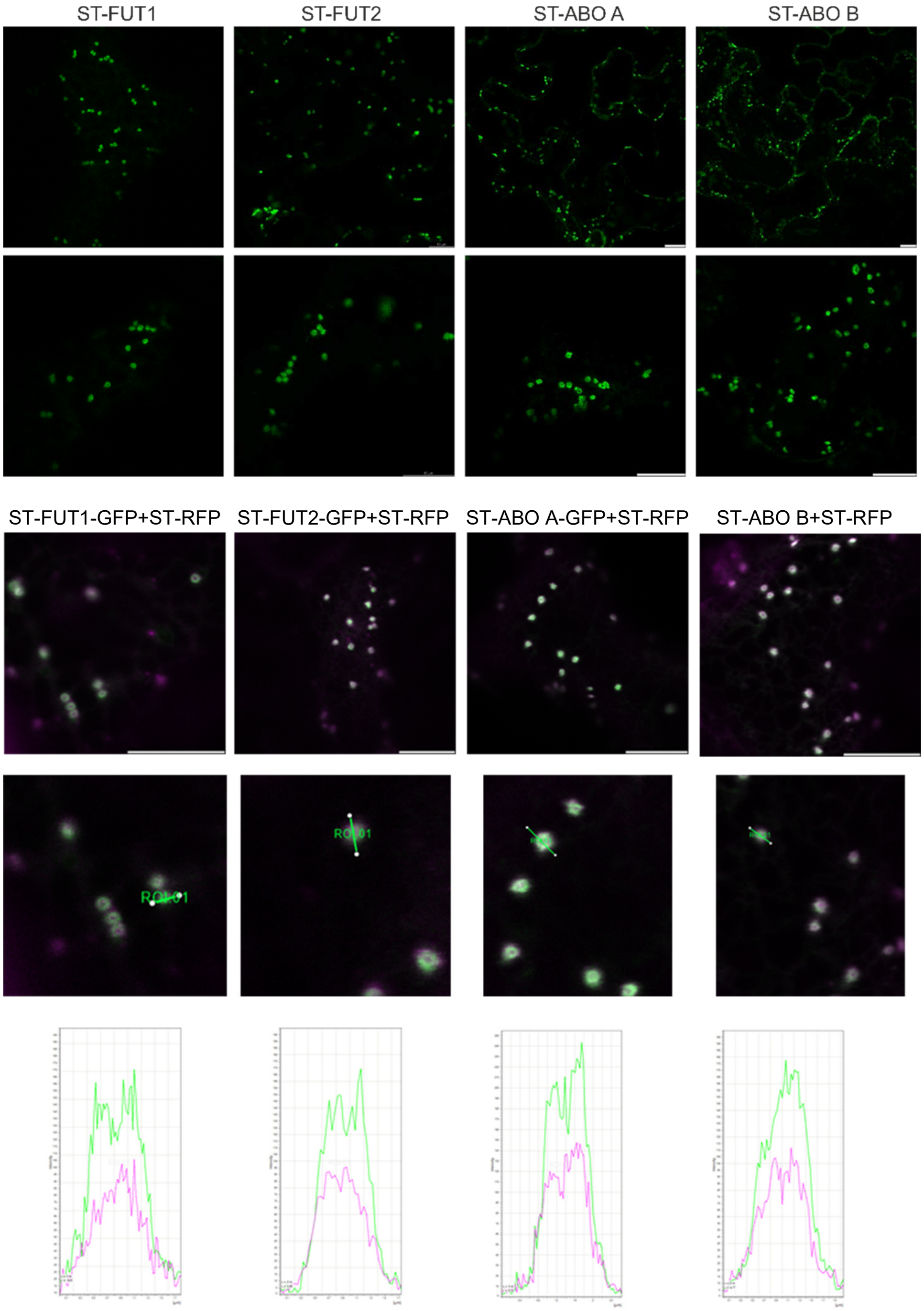

Figure S1. Glycosyltransferase fusion proteins localize to the Golgi apparatus. Leaves of 5-week-old wild-type *N. benthamiana* were infiltrated with agrobacterium suspensions carrying the plasmids for protein expression with an OD_{600} of 0.1. Confocal images were acquired 48 h after infiltration on a Leica SP5 confocal microscope (Leica Microsystems) as described previously (Strasser et al., 2007). Scale bars = 10 µm. Co-localization was done with the *trans*-Golgi marker ST-RFP.

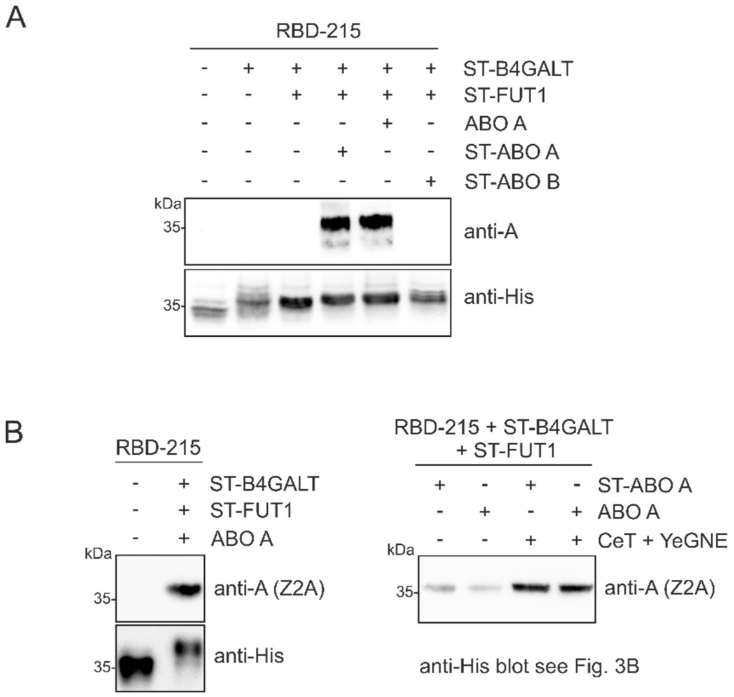

Figure S2. ABO and ST-ABO A display comparable activity when transiently co-expressed *in N. benthamiana* leaves. (A) RBD-215 was transiently expressed in the glycoengineered *N. benthamiana* line ΔXT/FT together with the indicated glycosyltransferases. 3 days-after infiltration, RBD-215 was purified from crude protein extracts using magnetic beads and subjected to SDS-PAGE and immunoblotting with blood group A (3-3A) or anti-His-tag antibodies. **(B)** Immunoblot analysis using the anti-A IgM antibody Z2A.

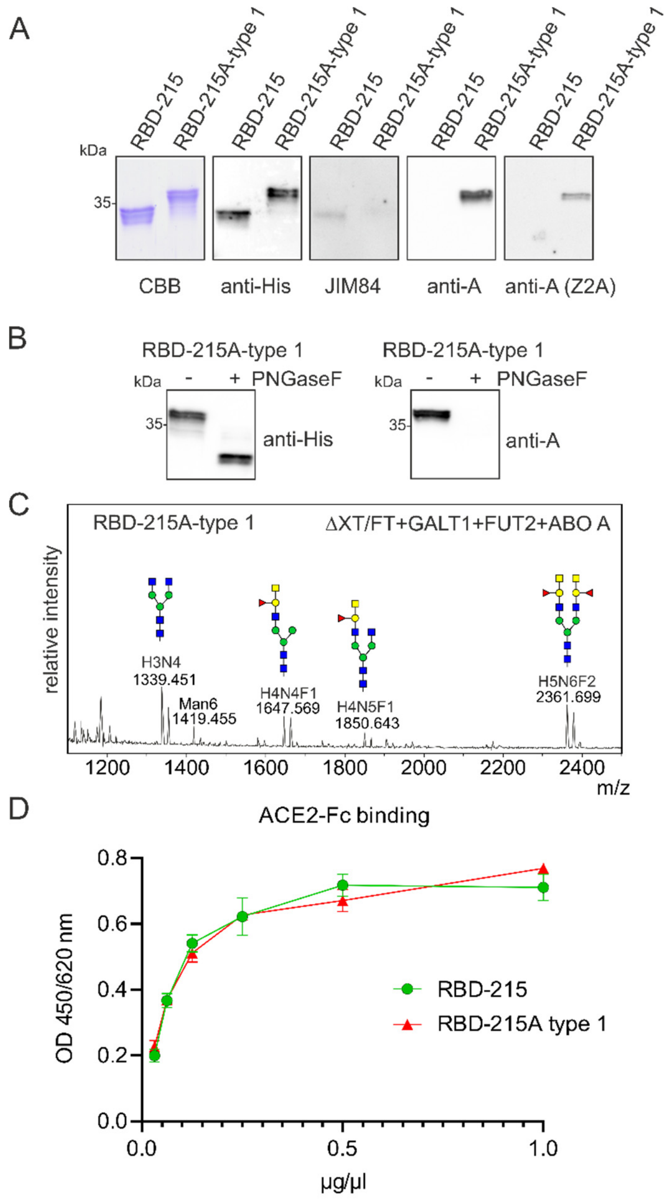

Figure S3. RBD-215 carrying blood group A type 1 antigens (RBD-215A-type 1) can be successfully produced in *N. benthamiana*. (A) RBD-215 was transiently expressed in the glycoengineered *N. benthamiana* line ΔXT/FT together with the glycosyltransferases and proteins for UDP-GalNAc formation and Golgi transport (GALT1, ST-FUT2 and ST-ABO A, YeGNE and CeT). 3 days-after infiltration, RBD-215 was IMAC purified from the apoplastic fluid and subjected to SDS-PAGE (CBB: Coomassie brilliant blue staining) and immunoblotting with the indicated antibodies. JIM84 binds to the Lewis A epitope (Strasser et al., 2007); anti-A, 3-3A IgG antibody; anti-A (Z2A), Z2A IgM antibody. **(B)** PNGase F digestion of RBD-215A-type 1 carrying the blood group A antigen at N-glycans. **(C)** MS spectrum of RBD-215A-type 1 N-glycans. 5 µg purified RBD-215A-type 1 was pepsin digested. N-glycans were subsequently released from glycopeptides using PNGase A, purified and analyzed by MALDI-TOF-MS. **(D)** ACE2-Fc binding ELISA. Binding of different concentrations of IMAC-purified RBD-215 and RBD1-215A-type 1 to plates coated with ACE2-Fc. Values represent the mean ± SD (n = 3).

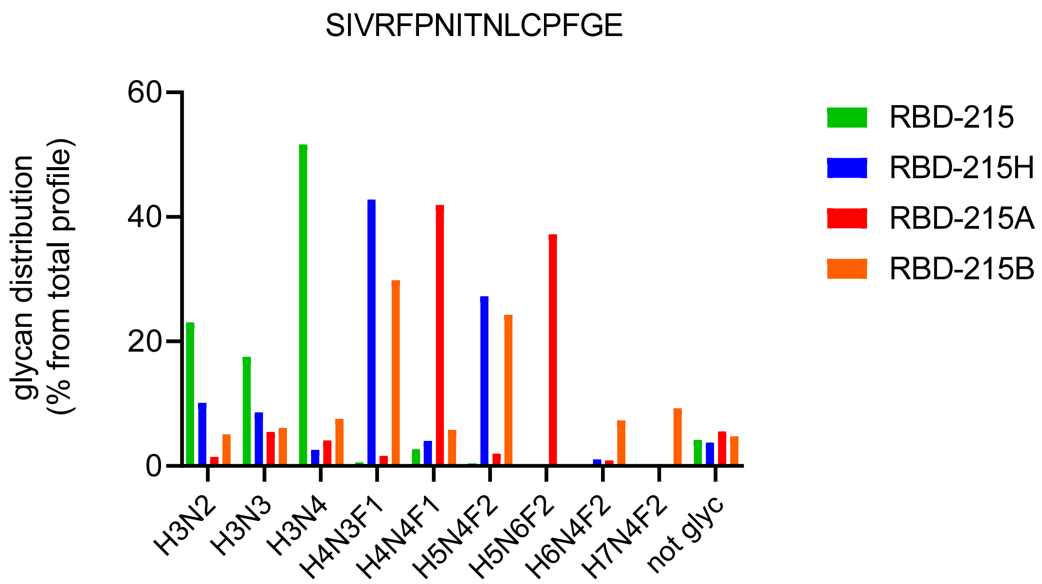

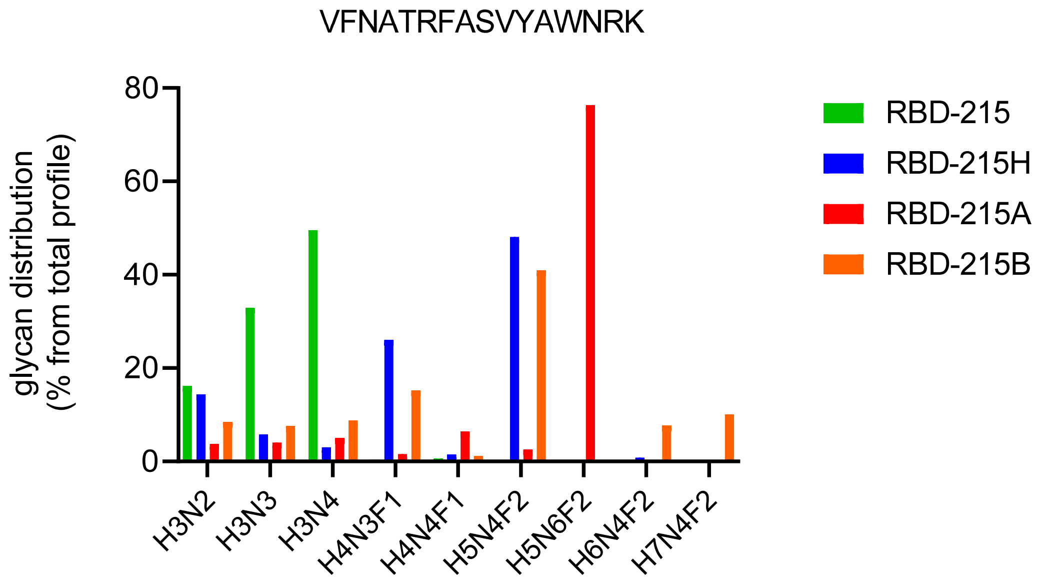

Figure S4. Quantification of RBD-215 glycopeptides. Manual glycopeptide searches were made using DataAnalysis 4.0 (Bruker). For the quantification of the different glycoforms the peak areas of EICs (Extracted Ion Chromatograms) of the first four isotopic peaks (all detected charge states) were summed, using the quantification software Quant Analysis 2.0 (Bruker). Non-glycosylated peptide (not glyc) was only detected for the peptide containing the NIT site.

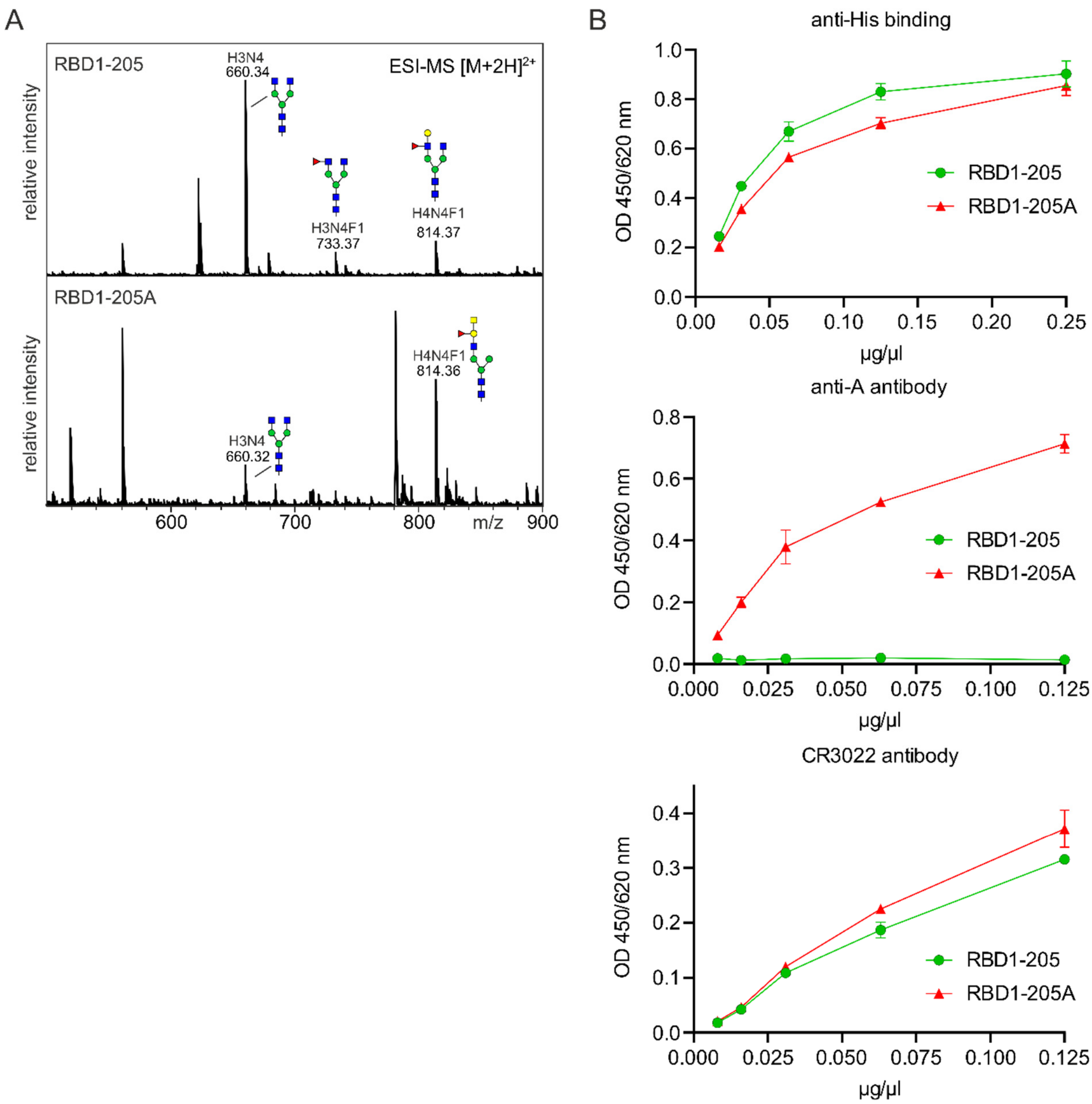

Figure S5. Mass spectrometry (MS)-analysis of RBD1-205 carrying blood group A type 2 N-glycans. (A) ESI-MS analysis of N-glycans released from RBD1-205 and RBD1-205A glycopeptides. **(B)** RBD1-205 and RBD1-205A were coated and ELISA was carried out with different concentrations of antibodies against the His-tag, the 3-3A antibody against anti-blood group A antigen structures and CR3022, a conformation-dependent antibody that binds to the spike protein from SARS-CoV-1 and SARS-CoV-2. Values represent the mean ± SD (n = 3).

Table S1. MFI values showing the binding of IgG from sera to RBD-215 glycoforms.

RBD negative									
		Median MFI					Median ΔMFI		
**Blood group**	**n=**	**RBD-215**	**RBD-215H**	**RBD-215A**	**RBD-215B**	**RBD-HEK**	**RBD-215H / RBD-215**	**RBD-215A / RBD-215**	**RBD-215B / RBD-215**
**0**	15	1452	1165	29384	3722	1200	0,80	20,24	2,56
**A**	14	1081	985	1023	1297	675	0,91	0,95	1,20
**B**	9	1248	1129	4874	1020	887	0,90	3,91	0,82
**AB**	6	1040	906	726	680	539	0,87	0,70	0,65
**RBD positive**									
		Median MFI					Median ΔMFI		
**Blood group**	**n=**	**RBD-215**	**RBD-215H**	**RBD-215A**	**RBD-215B**	**RBD-HEK**	**RBD-215H / RBD-215**	**RBD-215A / RBD-215**	**RBD-215B / RBD-215**
**0**	28	46086	38839	47365	32324	49954	0,84	1,03	0,70
**A**	32	35375	28866	27032	30843	35086	0,82	0,76	0,87
**B**	10	32624	26473	32803	28416	35956	0,81	1,01	0,87
**AB**	9	52669	45832	45336	49049	58377	0,87	0,86	0,93
